# Intent to Change Sun-Protective Behaviors Among Hispanic People After a UV Photoaging Intervention: Cohort Study

**DOI:** 10.2196/33339

**Published:** 2022-01-25

**Authors:** Levi N Bonnell, Ngozi Obi, Kimberly Miller, Sophia Hu, Robert Dellavalle, Myles Cockburn

**Affiliations:** 1 University of Colorado Cancer Center Aurora, CO United States; 2 Department of Dermatology University of Colorado Anschutz Medical Campus Aurora, CO United States; 3 Department of Clinical Preventive Medicine Keck School of Medicine of University of Southern California Los Angeles, CA United States; 4 University of Colorado School of Medicine University of Colorado Anschutz Medical Campus Aurora, CO United States

**Keywords:** risk assessment, sun safety, intention to change, sun exposure behavior, melanoma, Hispanic, sun damage, skin cancer

## Abstract

**Background:**

Mortality rates from melanoma are higher among Hispanic populations than non-Hispanic White (NHW) populations. Interventions to improve sun safety are needed. The Reveal Imager is a camera that uses standard cross-polarized flash photography to record surface and subsurface skin conditions.

**Objective:**

This study aims to determine the intervention’s effectiveness in increasing awareness of sun damage and exposure reduction between Hispanic and NHW populations.

**Methods:**

A cohort of 322 participants, aged ≥18 years, were recruited from community events in 2018. Baseline information was collected on demographics, sun exposure, and perception of risk factors. A facial image was then captured using the Reveal Imager. The results were explained and counseling on sun safety was given, followed by filling out an immediate postimage survey. Chi-square tests, analysis of variance, Wilcoxon signed-rank test, McNemar tests, and multivariable logistic regression were used.

**Results:**

At follow-up, 125 of 141 (89%) Hispanic participants reported that viewing the UV photoaged image influenced intent-to-change sun protection behaviors, compared to 88 of 121 (73%) NHW participants (odds ratio 2.9, 95% CI 1.5-5.6). Of 141 Hispanic participants, 96 (68%) reported that they intended to increase sunscreen use, compared to only 41 of 121 (34%) NHW participants (*P*<.001).

**Conclusions:**

We demonstrated an application of Reveal Imager for education and risk assessment. The Reveal Imager was especially helpful in motivating intention to change sun exposure among Hispanic populations.

## Introduction

Skin cancer is the most common malignancy in the United States, outnumbering all other cancers combined [1]. Although cutaneous cancers are uncommon in Hispanic people in the United States, mortality rates are much higher compared to non-Hispanic White (NHW) people [2]. These discrepant outcomes may be attributed to late detection and biologically more aggressive tumors [2-6].

Numerous studies suggest that Hispanic people differ in their perceptions of skin cancer risk compared to their NHW counterparts [3,7-10]. Hispanic populations perceive themselves to be at a low-risk for skin cancer due to their darker skin tone and lack of family history, and therefore are less likely to undertake sun-protective measures [10]. Buster et al [8] found that Hispanic people were more likely to believe they were unable to lower their skin cancer risk. Nonetheless, late-stage melanoma rates continue to rise in Hispanic populations [11]. The Hispanic population in the United States continues to grow, increasing the magnitude of this disease [5]. The lower prevention rates and poorer prognosis among the Latinx population necessitates interventions to increase awareness of skin cancer burden among this population.

The pattern of UV exposure is correlated with the development of different types of cutaneous melanoma, basal cell carcinoma (BCC), and suamous cell carcinoma (SCC). Overall, melanoma is correlated with long-term, intermittent UV exposure, BCC was found to depend on intensive sunlight exposure earlier in life before adulthood, and SCC was related to prolonged and persistent UV exposure over a period of decades [12]. Within melanoma, superficial spreading melanoma and nodular melanoma are associated with a history of sunburns and intermittent UV exposure in healthy young patients. In contrast, chronic lifetime sun damage increased the risk of developing lentigo maligna melanoma [13]. One case control study showed a correlation between multiple lifetime sunburns from UV exposures to increased incidences of superficial spreading melanoma but no link with lentigo maligna melanoma [14].

The increasing rates of skin cancers and mortality in Hispanic populations, the majority of which is SSM [15], makes protection against UV rays an issue of paramount importance [13,14]. Diligent UV protection is well known for its efficacy in preventing skin cancer occurrences [12]. However, in one study, although Hispanic adolescents reported engaging in sun protection behaviors, they were found to have higher rates of sunburns compared to national estimates for NHW children [16]. Thus, more efforts are needed to educate the Hispanic population and disseminate information on sun protection. Educational interventions geared toward sun protection are critical to early detection and prevention of future skin cancer–related mortalities [17].

In this study, we sought to compare the effectiveness of the Canfield Reveal Imager (UV photoaged facial imager) on intent-to-change sun-protective behaviors between Hispanic and NHW populations. We further characterized the Hispanic people who intended to change sun protection behaviors.

## Methods

### Study Population and Procedures

In this prospective cohort study, we recruited 322 adults (≥18 years of age) from 9 community events in Denver, Colorado from May 2018 through March 2019, primarily in the winter and summer seasons. Participants were recruited from diverse community and health promotion events ranging from cancer benefits, campus wellness fairs, to consulate events. Attendees of the event were introduced to the UV photoaged facial imager, given a brief description of the study, and offered an opportunity to participate. Informed consent was obtained by all participants, and the study was approved by the Colorado Multiple Institutional Review Board.

Data were collected at two time points: (1) baseline, immediately before the photoaged image, and (2) follow-up, immediately after the photoaged image, typically within 30 minutes of each other. All participants completed the baseline questionnaire that assessed demographic information, sun exposure history (both in childhood and in the past year), sun protection behaviors, perceptions of tanning, and perceived risk of skin cancer.

**Figure 1:**
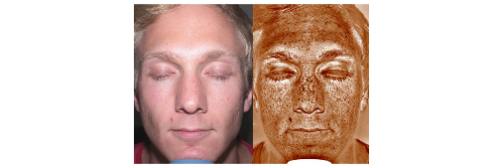
Standard-light facial photograph (left); cross-polarized filter photograph (right).

After the baseline questionnaire was completed, participants had a UV photoaged facial image taken and shown to them (Figure 1). The investigators consisted of a medical resident, medical students, and research coordinators who interpreted the images, answered participant questions, and provided sun protection education. Participants were then asked to complete a postimaging follow-up questionnaire that assessed perceptions of tanning, perceived risk of skin cancer, and intent-to-change sun protection behaviors after seeing the UV photoaged facial image.

### Description of the Intervention (UV Photoaged Image)

The Canfield Reveal Imager (UV photoaged facial imager) is a camera that uses standard white light and cross-polarized flash photography to record surface and subsurface skin conditions, capturing two images in quick succession. The crossed-polarizing filter reduces skin surface reflections and allows visualization of skin changes and damages (eg, brown pigmentation, wrinkles, and lines) and provides immediate visual feedback to the individual, demonstrating the harm caused by chronic sun exposure. The UV photoaged facial image can be used as a form of fear appeals educational intervention for skin cancer. The two images are juxtaposed on the screen for visualization and the education of participants (Figure 1).

### Education

Study team members were trained on by the principal investigator on interpreting the UV photos and delivering education to the participants. Participants were shown reference photos from the UV Reveal Camera of individuals with varying levels of sun damage visualized prior to seeing their own UV photos. This prior knowledge provided context for participants to self-assess the amount of sun damage they accumulated relative to standard controls. Verbal feedback was provided by the medical team by pointing out specific areas of sun damage (brown spots) visualized on the UV Reveal Camera. Any further questions were answered.

### Measures

Sun protection behaviors, perceptions of tanning, and risk perception of skin cancer was based on a subset of items from the Sun Protection Awareness Questionnaire. The following are the measured items:

- Sunscreen use (preimage only): Frequency of sunscreen use in the past 12 months was assessed from Always to Never. The sun protection factor of the sunscreen was recorded. If sunscreen was never used, open text responses of reasons why sunscreen was not used were recorded.
- Sun protection behavior (preimage only): Childhood and adult sun protection behaviors were assessed using three questions. Two questions asked about protective clothing worn during childhood and adulthood. Age at first deep tan was also asked.
- Perceptions of tanning: Perceptions of tanning were measured with three questions asking participants to measure their agreement from untrue to very true on statements about the importance of tanning, if tanning increases attractiveness, and if the participant wanted to get a tan. For the analysis, these were dichotomized into untrue and somewhat untrue versus somewhat true and true.
- Risk perception of skin cancer: Risk perception of skin cancer was measured using three questions. Two asked participants to measure their level of agreement from untrue to very true to statements about current sun exposure and future risk of developing damaged skin and skin cancer. The second question asked participants to compare their risk of developing skin cancer to an average person of similar age and sex, with answers ranging from “I am at much less risk than others” to “I am at much greater risk than others.” For the analysis, these were dichotomized into untrue and somewhat untrue versus somewhat true and true.

Both the baseline and follow-up questionnaires were completed in-person on a paper. The primary outcome of this study was intent-to-change sun protection behaviors immediately after seeing the UV photoaged image. This variable was originally collected with 3 levels (yes/no/unsure) but was dichotomized as yes versus no/unknown. The primary independent variable was ethnicity (Hispanic vs NHW). Secondary outcomes included change of pre- to postimage perceptions of tanning and risk perception of skin cancer.

### Statistical Analysis

The analysis included participants that identified as Hispanic or NHW (n=278), which comprised 86% of the total sample (N=322). We excluded other races because our primary research question focused on Hispanic individual’s sun protection behaviors compared to NHW individuals, and the sample size was small for other races. We excluded records with a missing pre- or postimage date. Missing data analysis using analysis of variance for continuous variables and Pearson chi-square tests for categorical variables were conducted on demographic variables to determine if any differences existed by ethnicity.

Descriptive statistics were performed on baseline demographic information and sun exposure information. The proportion of individuals who intended to change sun protection behaviors was compared using Pearson chi-square test. We compared the coefficient on the predictor from a mixed model with no fixed effect covariates to that from a model with a single covariate. If the coefficients differed by >10%, the covariate was included in the full multivariate analysis. Mixed effects logistic regression was used to assess the relationship between ethnicity and intent-to-change sun protection behaviors. Ethnicity was included in the model as the main effect, and education and age were included in the model as covariates. Location of community event that the interview took place was dichotomized (health event vs not health event) and included in the model as a random intercept. In a post hoc analysis, we included the final model stratified by season (winter vs summer) to investigate if this relationship varied by the season in which the data were collected.

Changes in tanning and skin cancer perception from pre- to postimage were compared using McNemar test and Wilcoxon signed-rank tests for ordinal, repeated data. Variables that changed the coefficient more than 10% were included in the final multivariable model. A mixed effects linear model was used to assess main effects of the intervention, estimating mean change in perception of tanning and risk of skin cancer from pre- to postimage. Ethnicity, education, and age were included in the model as fixed effects. An alpha criterion of *P*<.05 was used. All tests were 2-tailed. Statistical analyses were performed using Stata version 15 (StataCorp). This study was approved by the Colorado Multiple Institutional Review Board.

## Results

### Description of Cohort

We recruited 278 Hispanic and NHW participants from 9 community events. Of the 278 participants, 262 (94%) completed the follow-up questionnaire. Comparisons of baseline information by ethnicity are described in Table 1. At baseline, compared to NHW participants, Hispanic participants were younger, less educated, more likely to work outdoors, had fewer self-reported past diagnoses of skin cancer, and were less likely to use sunscreen in the past 12 months. Furthermore, Hispanic participants’ perceived risk of developing skin cancer was lower; they were more likely to think a tan made them look attractive and were more likely to want a tan. By contrast, NHW participants were more likely to think they needed to cut down on tanning and felt guilty about tanning. No differences by ethnicity were observed by sex, perceptions on developing wrinkles, skin damage, skin cancer from sun exposure, importance of a tan, or use of tanning beds in the last 12 months. Missingness analyses found there were no significant differences between those who did not complete the pre- and postimage surveys by ethnicity, age, sex, or intent-to-change sun protection behaviors.

**Table 1:** Baseline characteristics of cohort stratified by ethnicity (n=278).

		Hispanic (n=150)	Non-Hispanic White (n=128)	*P* value
**Baseline demographic and clinical information**				
	Age (years), mean (SD)	40.7 (11.4)	44.9 (15.2)	.01
	Sex (male), n (%)	60 (40.3)	43 (33.6)	.25
	Education (high school graduate or less), n (%)	98 (67.1)	19 (15.2)	<.001
	Occupation (outdoor), n (%)	39 (26.9)	9 (7.3)	<.001
	Previous skin cancer diagnosis (yes), n (%)	9 (6.1)	22 (17.7)	.002
	Season of event (summer), n (%)	10 (6.7)	115 (90)	<.001
**Sun protection behaviors and perceived risk of skin cancer, n (%)**				
	Sunscreen use (always or usually)	42 (28.2)	80 (64.0)	<.001
	Perceived risk of skin cancer	24 (64.9)	10 (15.6)	<.001
	Too much sun now may lead to wrinkles and skin damage	21 (14.0)	12 (9.4)	.24
	Too much sun now may lead to skin cancer	17 (11.3)	13 (10.2)	.75
**Perceptions of tanning, n (%)**				
	Good tan makes me more attractive (yes)	110 (73.3)	50 (39.1)	<.001
	Important to have a tan (yes)	112 (74.7)	86 (67.2)	.17
	Want to get a tan (yes)	114 (76.0)	74 (57.8)	.001
	Used a tanning bed in last 12 months (yes)	19 (12.7)	10 (7.8)	.19
	Felt you needed to cut down on tanning (yes)	18 (12.2)	24 (20.9)	.06
	People criticized you for tanning (yes)^a^	7 (7.8)	2 (1.8)	.31
	Felt guilty about tanning (yes)	10 (7.0)	24 (21.8)	.001

^a^Fisher exact test.

### Intent to Change: Primary Outcome

At follow-up, 213 of 262 (81%) participants reported that viewing the UV photoaged image influenced an intent-to-change sun protection behaviors. However, this differed by ethnicity. Of 141 Hispanic participants, 125 (89%) reported a likelihood of change compared to 88 of 121 (73%) NHW participants (odds ratio 2.9, 95% CI 1.5-5.6). Demographic and clinical information, sun protection behaviors, perceived risk of skin cancer, and perceptions of tanning were not associated with intent-to-change sun protection behaviors. However, these were included in the multivariable model as covariates based on clinical importance. After adjusting for age, education (high school graduate or less vs some college or more), perceiving a tan was more attractive, tanning bed use, and normal sunscreen use, Hispanic participants were significantly more likely to have an intent-to-change sun protection behaviors compared to NHW participants (adjusted odds ratio [aOR] 4.04, 95% CI 1.6-10.4; Table 2). In post hoc analysis, Hispanic participants were more likely to have an intent-to-change sun protection behaviors compared to NHW participants in both summer (aOR 3.28, 95% CI 0.5-25.3) and winter seasons (aOR 4.54, 95% CI 1.1-18.2), although not significantly in summer due to reduced sample sizes.

The most common sun protection behavior changes that participants intended to implement were increases in sunscreen use (134/262, 51%), to start wearing protective clothing like hats (39/262, 15%), and reapplication of sunscreen (26/262, 10%). These sun protection behaviors also varied by ethnicity. Of the 141 Hispanic participants, 96 (68%) reported that they intended to increase sunscreen use, compared to only 41 of 121 (34%) NHW participants (*P*<.001). More Hispanic participants also reported the intention to reapply sunscreen more often, while NHW participants were more likely to report the intent-to-increase wearing protective clothing like hats, but neither of these differences were statistically significant.

Hispanic participants that intended to change their sun protection behaviors after viewing the UV photoaged image (125/141) trended toward being younger (*P*=.09), working indoors (*P*=.13), and having a high school degree or less (*P*=.07) than Hispanic participants that did not intend to change their sun protection behaviors. Of 122 Hispanic participants, 110 (90%) with low perceived risk of skin cancer at baseline intended to change their sun protection after seeing the UV photoaged image behaviors, compared to only 15 of 19 (79%) with high-perceived risk (*P*=.11). Sex, previous skin cancer diagnosis, sunscreen use, perceived risk of wrinkles, and perceptions of tanning did not differ by intent-to-change sun protection behaviors among Hispanic participants.

**Table 2:** Univariate and multivariable relationships of risk factors and intent to change sun protection behavior (m=262).

			Intent to change sun protection behaviors		
			OR^a^ (95% CI)		aOR^b^ (95% CI)
**Baseline demographic and clinical information**					
	Ethnicity (Hispanic)	2.9 (1.5-5.6)^c^		4.0 (1.6-10.4)^c^	
	Mean age (SD)^d,e^	1.0 (0.96-1.0)		N/A^f^	
	Sex (male)	1.2 (0.61-2.3)		N/A	
	Education (high school graduate or less)^e^	0.9 (0.49-1.7)		N/A	
	Occupation (outdoor)	1.8 (0.85-3.8)		N/A	
	Previous skin cancer diagnosis (yes)	0.7 (0.30-1.6)		N/A	
**Sun protection behaviors and perceived risk of skin cancer**					
	Sunscreen use (always or usually)	1.4 (0.73.2.5)		N/A	
	Compared with the average person, risk of skin cancer	1.2 (0.49-2.9)		N/A	
	Too much sun now may lead to wrinkles and skin damage	0.7 (0.22-2.0)		N/A	
	Too much sun now may lead to skin cancer	1.0 (0.37-2.5)		N/A	
**Perceptions of tanning**					
	Good tan makes me more attractive (yes)^e^	0.6 (0.34-1.19)		N/A	
	Important to have a tan (yes)	0.9 (0.44-1.7)		N/A	
	Want to get a tan (yes)	1.0 (0.51-1.9)		N/A	
	Used a tanning bed in last 12 months (yes)^e^	0.4 (0.08-1.5)		N/A	
	Felt you needed to cut down on tanning (yes)^g^	Undefined		N/A	
	People criticized you for tanning (yes)^g^	Undefined		N/A	
	Felt guilty about tanning (yes)	1.8 (0.58-5.3)		N/A	

^a^OR: odds ratio.

^b^aOR: adjusted odds ratio.

^c^*P*<.001.

^d^Continuous variable.

^e^Included in final multivariable model.

^f^N/A: not applicable.

^g^Zero counts lead to undefined analysis.

### Pre- to Postimage Changes: Secondary Outcomes

#### Perceptions of Tanning

Perceptions of tanning did not change significantly from pre- to postimage. Hispanic participants had perceived decrease in “importance of tanning” (*β*=–.06; *P*=.87), “attractiveness from tanning” (*β*=–.33; *P*=.35), and wanting to get a tan (*β*=–.13; *P*=.73) from pre- to postimage compared to NHW participants. These results, although not statistically significant, indicate that perception of tanning changed more for Hispanic participants than NHW participants and moved in the expected direction.

#### Risk Perception of Skin Cancer

Risk perception of skin cancer did not change from pre- to postimage. Hispanic participants had a perceived decrease in “risk of developing skin cancer compared to an average person of similar age and sex” (*β*=–.15; *P*=.78) and “risk of cancer” (*β*=–.30; *P*=.61), while an increase of perceived “future skin damage” (*β*=.45; *P*=.50) was observed.

## Discussion

In this study, we demonstrated the feasibility of using the Canfield Reveal Imager to motivate intent-to-change sun protection behaviors among NHW and Hispanic populations. We also showed the efficacy of the modified Sun Protection Awareness Questionnaire for education, risk assessment, and improvements in sun safety behaviors. Our study found that showing the damaging effects of the sun on skin, in addition to education provided by a medical provider can motivate intent-to-change behaviors in Hispanic populations who traditionally perceive themselves to be at lower risk to developing skin cancer. An image demonstrating photo damage along with verbal sun protection education by medical personnel was especially helpful among Hispanic participants with a baseline low-perceived risk of skin cancer.

Fear appeals is a strategy used in public health to change behaviors. Public health campaigns such as antismoking, antialcohol, and hypertension awareness have used the fear appeals methods [18]. However, most of the literature suggests that fear appeals are ineffective in motivating changes in behavior [18-22]. On the contrary, the target population may feel threatened but are still not convinced of the effectiveness of the alternative behavioral modification. Indeed, they may become more defensive and oriented toward avoidance of the health-promoting messages rather than actions toward adoption [23]. The extended parallel process model suggests that the impact of fear appeals is most effective when they include both a threat emphasizing severity and susceptibility, as well as recommended actions that reinforce self-efficacy [19,24,25]. In a recent randomized controlled trial (RCT), UV skin damage visuals generated greater fear than other visuals (sun burn, mole removal, and photoaging), resulting in increased sun safe behaviors [26]. In a study of Facebook skin cancer prevention groups, fear was the most used persuasive appeal [27]. Similar to the RCT, we found that intent-to-change sun protection behaviors after a fear appeals intervention was high, especially among Hispanic participants.

Hispanic participants in our sample may have been especially responsive to the UV photoaged facial image, a type of fear appeals intervention, because their perceived risk of skin cancer was lower at baseline. More studies are needed to determine if this finding is generalizable. Further, visualizing the actual skin damage caused by chronic sun exposure when there is still time to act could potentially influence intention-to-change sun protection behaviors.

Despite evidence of a higher intent-to-change among Hispanic participants, perceptions of the risks of tanning and skin cancer did not change from pre- to postviewing the image. This finding suggests that the Reveal Imager has the potential to help promote sun awareness but not necessarily increase knowledge around the risks of tanning and skin cancer.

Our study also demonstrates that community-based screening programs held at large events provide an opportunity to identify a substantial number of people who could benefit from sun protection education. Importantly, our study has implications for future efforts to educate the public about minimizing skin cancer risk. Educational endeavors may be particularly efficacious if used in combination with fear appeals and a visual component with direct involvement of the participant. Given the highly preventable nature of the disease, successful education and implementation of sun-protective measures may decrease new incidences of skin cancer over time, perhaps leading to substantial shifts in epidemiological trends in the future.

Strengths of this study include strong representation from NHW and Hispanic populations from various neighborhoods around the Denver Metro Area, measurement of attitudes toward both sun-exposing and sun-protective behaviors, and being among the first studies to use fear appeals as an intervention to target changes in sun-protective behaviors among Hispanic people.

There were also limitations to this study. First, there is likely self-selection bias as people who attend health and wellness fairs and cultural events are likely more health conscious or may be more open to health behavior prompts than those who do not attend. Second, most of the participants were women and all lived in Denver, Colorado, which reduces the generalizability of these results. However, because we recruited from substantially different neighborhoods, we think the results are at least generalizable to the Denver Metro Area and possibly to other diverse cities. Third, other booths at the events presented information on sun protection behaviors and skin cancer awareness. The proximity of this information may have contaminated our results. Fourth, *Hispanic* is a heterogeneous category, and heritage subgroups may differ from one another; there are other unmeasured cultural variables (nativity, acculturation, language preference). Further, there is considerable variety of skin pigmentation among those who identify as Hispanic, and this may be associated with sun protection habits. However, we did not collect information on pigmentation. Fifth, the questionnaire and education were only offered in English. Finally, in addition to the small sample size, there was a large difference in education levels between NHW and Hispanic populations.

Compared to NHW participants, Hispanic participants are more likely to be diagnosed in later stages when the cancers are more difficult to treat and survival rates are lower [28,29]. This motivated us to compare an educational intervention that has worked among NHW participants as a potential educational intervention for sun protection behavior to Hispanic participants [30]. We found that Hispanic respondents were more likely to have intent-to-change sun protection behavior compared to NHW participants after viewing the UV photoaged facial image. The virtue of the UV photoaged image is that it provides an immediate, easily comprehensible measurement of personal risk and individual assessment of sun-induced skin damage that could otherwise remain invisible to the naked eye, especially among Hispanic people, who perceive themselves as being at lower risk for skin cancer. It is important to adopt different forms of awareness for the primary prevention of skin cancers, especially in populations at risk. Although the use of polarized flash photography is no longer innovative, it can be a useful tool to raise awareness, especially among vulnerable populations.

## Abbreviations

aOR: adjusted odds ratio
BCC: basal cell carcinoma
NHW: non-Hispanic White
RCT: randomized controlled trial
SCC: squamous cell carcinoma
